# Global transcriptional response of pig brain and lung to natural infection by Pseudorabies virus

**DOI:** 10.1186/1471-2180-9-246

**Published:** 2009-12-01

**Authors:** JF Yuan, SJ Zhang, O Jafer, RA Furlong, OE Chausiaux, CA Sargent, GH Zhang, NA Affara

**Affiliations:** 1 Key Lab of Agricultural Animal Genetics, Breeding and Reproduction of Ministry of Education, HuaZhong Agricultural University, 430070, PR China; 2 Department of Pathology, University of Cambridge, Cambridge CB2,1QP, UK; 3 College of Veterinary, South China Agricultural University, 510642, PR China

## Abstract

**Background:**

Pseudorabies virus (PRV) is an alphaherpesviruses whose native host is pig. PRV infection mainly causes signs of central nervous system disorder in young pigs, and respiratory system diseases in the adult.

**Results:**

In this report, we have analyzed native host (piglets) gene expression changes in response to acute pseudorabies virus infection of the brain and lung using a printed human oligonucleotide gene set from Illumina. A total of 210 and 1130 out of 23,000 transcript probes displayed differential expression respectively in the brain and lung in piglets after PRV infection (p-value < 0.01), with most genes displaying up-regulation. Biological process and pathways analysis showed that most of the up-regulated genes are involved in cell differentiation, neurodegenerative disorders, the nervous system and immune responses in the infected brain whereas apoptosis, cell cycle control, and the mTOR signaling pathway genes were prevalent in the infected lung. Additionally, a number of differentially expressed genes were found to map in or close to quantitative trait loci for resistance/susceptibility to pseudorabies virus in piglets.

**Conclusion:**

This is the first comprehensive analysis of the global transcriptional response of the native host to acute alphaherpesvirus infection. The differentially regulated genes reported here are likely to be of interest for the further study and understanding of host viral gene interactions.

## Background

Pseudorabies virus (PRV), is a member of the alphaherpesvirus subfamily and has multiple closely related family members, such as the herpes simplex virus1 (HSV-1), varicellovirus (VZV), avian herpes viruses, bovine herpesviruses (BHV-1), equine herpesviruses (EHV-1 and EHV-4), feline herpesvirus type 1 and canine herpesvirus type [1,2]. Thus PRV has served as a useful model organism for the study of herpesvirus biology[1]. Owing to its remarkable propensity to infect synaptically connected neurons, PRV is also studied as a "live" tracer of neuronal pathways[1]. Finally, while vaccination strategies to eradicate PRV in the United States and Europe have shown great progress, they fail to eradicate completely viral infection from a population. Thus outbreaks in swine populations result in substantial economic losses. These include restrictions on animal movement and trade for affected countries, with disease and infection control measures increasing production costs owing to antibody testing, vaccination programs and extra labor.

Although PRV has been widely studied (especially its agricultural impact, its viral pathogenesis, its molecular biology, its use as a neuronal tracer, and in DNA vaccine exploration [1]) how the native host responds globally after infection with wild type PRV is still poorly understood. Clinically, infection in older pigs ranges from asymptomatic to severe respiratory disease but with limited mortality. Young piglets exhibit more serious clinical signs and often succumb to fatal encephalitis preceded by typical behaviors consistent with infection of the central nervous system. In recent years, microarray technology has proven useful to assess the cellular transcriptional responses to herpesvirus infections in human and mouse cell lines [3-5]. It has been used to study host gene expression after PRV infection of rat embryo fibroblasts [5], and the central nervous system (CNS) in rodent brain at various times post infection *in vivo *[6]. However few porcine genome-wide expression studies have been published. Most experiments have used 'in-house' cDNA arrays to study transcriptional events in pig tissues, such as the stress-genes related to early weaning of piglets [7]. The down side of these cDNA-based clone libraries is that the genes represented on the array are often very focused on a given biological system or process and lack a whole genome overview.

In this study, piglet samples were hybridized onto an Illumina Human Refset Chip (Illumina Inc. San Diego), corresponding to 23,000 transcript probes. This cross-species comparison potentially allows the study of the whole transcriptome. There are now porcine arrays available from commercial suppliers (e.g. Affymetrix and Qiagen), but these are not all representative of the entire pig genome and were not widely available at the time of this study. In the absence of a comprehensive species-specific array deeper interrogation of the pig gene complement was afforded by the use of the better annotated human geneset. Although the use of this approach can only be partially informative when there are no confirmed pig orthologues in the public databases, we have identified host cellular genes whose mRNA levels change during natural PRV infection of piglet brain and lung. The resulting data define key pathways of host-gene expression that characterize the host response to an acute central nervous system (CNS) and respiratory infection.

## Methods

### Experimental pigs and housing

The experimental animals were sourced from an outbreak of PRV that occurred in the farrowing house of a local commercial farmer due to a reduced level of protection via maternal antibody. Clinical signs were described as follows: suckling piglets were listless, febrile, and uninterested in nursing. Within 24 h of exhibiting these clinical signs, some piglets progressively developed indications of central nervous system infection including trembling, excessive salivation, lack of coordination, ataxia, and seizures. Infected piglets sat on their haunches in a "dog-like" position, lay recumbent and paddled, or walked in circles. The appearance of the dissected organs in selected piglets was typical of PRV infection: bleeding in meninges, oedema in the brain, bleeding spots in the lung and on the adenoids [1,8].

Three strict criteria were imposed for the selection of piglets included in this study: 1) piglets exhibited the typical clinical signs described above; 2) piglets exhibited the expected pathology, especially in brain and lung; 3) virus isolation, antibody identification or detection of viral antigen-positive tissues were used to confirm the organic infection by PRV, and diseases including Swine Fever (SF), Porcine Reproductive and Respiratory Syndrome Virus (PRRSV) and other potential bacterial infections which could be clinically and pathologically confused with PRV infection were excluded by viral antigen, antibody identification and PCR detection.

Six piglets aged from 2 to 4 days (commercial breed Landrace X Yorkshire) which were infected by PRV but not by the other tested diseases (see above) and 3 healthy piglets (not infected, and negative for all tests under the strict criteria used above), matched for age and breed from the same farm were used in this experiment. All experiments were carried out in strict accordance with accepted HuaZhong Agricultural University, China and governmental policies.

### Microarray experimental design

Total mRNA samples from the brains and lungs of the 3 normal piglets were pooled for the reference mRNA. Ten independent RNA samples (6 biological replicates for brain and 4 biological replicates of lung) from the 6 infected piglets were paired with the reference sample for hybridization on two-color microarrays. Using a dye-swap configuration, comparing each sample provides technical replicates to adjust for dye bias[9]. A total of 20 slides were used in this study.

### RNA purification

Total mRNA was prepared using Qiazol reagent (Qiagen, Crawley, West Sussex, UK) following the manufacturer's instructions. A second purification step was performed immediately post extraction on the isolated total mRNA using the RNeasy Midi kit (Qiagen Inc., Valencia, CA) and each sample was treated with DNase (20 U of grade I DNase; Roche, Lewes, UK) to remove any genomic contamination following the manufacturer's instructions. With a cut-off of 150 bp, 5S rRNA and tRNAs were removed from the samples by the columns, limiting interference in downstream experiments. RNA concentration and integrity were assessed on the Nanodrop ND-1000 spectrophotometer (Nanodrop, USA) and on the Agilent 2100 bioanalyzer system (Agilent Technologies, Palo Alto, CA), using an RNA 6000 Nano LabChip kit.

### SMART amplification and labeling of the samples

The extracted RNA was amplified using the SMART amplification protocol (BD Smart TM Amplification Kit, UK) and labeled with cy5 or cy3 using Klenow enzyme as described by Petalidis et al 2003 [10] with two modifications; (a) a constant number of 14 cycles was used, and (b) for the labeling step, 1 μL of Cy3 or Cy5-dCTP was used with 22 μL (250 ng) of second strand cDNA. The labeled products were purified using G50 columns, according to manufacturer's instructions (Amersham Biosciences, UK). Labeled samples were combined and precipitated for at least 2 hours at -20°C with 2 μL of human Cot-1 DNA, 1 μl PolyA (8 μg/μl), 1 μl yeast tRNA (4 μg/μl), 10 μl Na acetate (3 M, pH5.2) and 250 μl 100% ethanol.

### Microarray hybridization and scanning

The labeled product was re-suspended in 40 μL hybridization buffer (40% deionised formamide, 5 × SSC, 5 × Denhart's, 1 mM Na Pyrophosphate, 50 mM Tris Ph 7.4 and 0.1%SDS) and hybridized onto a microarray slide containing 23,000 human oligonucleotides (Illumina Inc. San Diego), printed in-house on to Codelink slides using a BioRobotics Microgrid II arrayer. After over-night hybridization of the slides at 48°C in a water bath, they were washed in 2 × SSC, 0.1 × SSC, 0.05% Tween 20, and 0.1 × SSC sequentially for 5 min each and scanned using an Axon 40001A scanner. Signal quantification was performed using Bluefuse software (2.0) (BlueGnome, Cambridge, UK).

### Analysis of the data

Data exported from Bluefuse was analyzed using the R package http://www.r-project.org/ library FSPMA [11], which is based on the mixed model ANOVA library YASMA [12]. Expression values in both channels were converted to log ratios and normalized by subtracting a M/A (i.e. log ratio/log amplitude) loess fit and adjusting the within-slide scale of the data. The ANOVA model used a nested design with spot-replication (1) as the innermost effect, nested inside biological replication (6 for brains; 4 for lungs), with dye-swap (2) as the outermost effect. Spot-replication was considered to be a random effect and biological replication and dye-swap fixed effects. Genes were considered to be up or down regulated, if the average channel log ratios relative to the control were found to be highly significantly different from zero, using a p-value threshold of 0.05. The p-values were calculated within the ANOVA model, using FSPMA's VARIETY option and a correction for multiple comparisons by false discovery rate. This analysis takes into consideration the variance across samples and excludes those genes with a high level of variance. We can, therefore, be confident that the smaller fold changes observed are real.

70-mer human oligonucleotide sequences from differentially expressed probe sets with a p-value < 0.01 were used to BLAST search pig sequences in the public databases http://www.ncbi.nlm.nih.gov/BLAST/ including Unigene and ESTs [13]. For matches to Unigene clusters, Homologene was used to indicate orthology to the human probe sets. With novel ESTs, pig data were matched against the human genomic and transcript database to confirm that the best matches were to orthologous sequences. Hits were considered to be reliable if there was a putatively orthologous match of 60-70 bp, and oligonucleotides with fewer matches, in the range of 50-59 bp, were also selected if p-values were significant in this study. Probe sets that could not be verified by BLAST as described above are not reported in this paper. Analysis of the signal intensity distribution of the cross-species hybridizations for both the lung and brain experiments showed a normal distribution similar to that obtained when homologous human RNA is hybridized to the chip. The proportion of the approximately 23K probes showing a signal greater than 100 signal value (i.e. above background) in the cross-hybridization is 22,300 from the 22,800 probes on the chip (~97%). The microarray data (accession number E-MEXP-2376) is available through ArrayExpress.

### Functional annotation of gene expression data

In order to understand the biological phenomena studied here and reduce the interpretive challenge that is posed by a long list of differentially expressed genes. Onto-Express was used to classify our lists of differentially regulated genes into functional profiles characterizing the impact of the infection on the two different tissues http://vortex.cs.wayne.edu/ontoexpress/[14]. Initial analysis used the non-filtered dataset, i.e., all differentially regulated probe sets against the full human oligonucleotide geneset. We then looked at differentially expressed probes (p-value < 0.01) identified from our microarray analysis, and statistical significance values were calculated for each category using the binomial test available in Onto-Express[15]. This makes no assumptions about those probesets with good matches to known pig sequences. However, only those probesets for which we could confidently assume orthology are reported in the tables in this paper. Here we present categories of gene ontology based on a maximum pairwise p-value of 0.05 for the "biological processes". To gain a better understanding of the gene interactions (pathways) involved in the disease, Pathway-Express was also applied to our data. In order to quantify the over/under representation of each category, the library composition has been taken into account in the presentation of the results.

### Quantitative RNA analyses using real-time PCR methodology (qRT-PCR)

Quantitative real-time reverse transcriptase polymerase chain reaction (qRT-PCR) analysis using SYBR green and selected primers was carried out following the manufacturer's protocol (QIAGEN, QuantiTect SYBR Green RT-PCR) to confirm the microarray results. All probes and primers were designed using Express Primer 3 software developed by the Whitehead Institute for Biomedical Research. The nucleotide sequences of selected genes were obtained from GenBank, and the primer information is shown in table 1. *PSMD2 *(primers kindly provided by Ms Gina Oliver and Dr Claire Quilter) was selected for use as the reference gene because it was previously shown to be a good control for pig brain (personal communication from Ms Gina Oliver and Dr Claire Quilter) and was also shown to be one of the most constant housekeeping genes in a human tissue study. Quantitative RT-PCR was performed on 300 ng RNA equivalents in 25 μL/reaction/well on an Icycler (Bio-Rad Laboratories Ltd, USA) (50°C for 60 min; 95°C for 15 min; 40 cycles of 95°C for 15 sec, 58°C for 30 sec and 72°C for 30 sec). For each gene reactions were performed in triplicate to allow statistical evaluation of the data. The average Ct (threshold cycle) was used for the analysis. Relative expression levels were calculated by using the 2^-(ΔΔCt) ^method as previously described [16].

**Table 1:** validation of array data by real-time PCR

			Microarray data		qRT-PCR data	
			
Gene name	Pig homologene	Primer sequences (5'-3')	Brain (n-fold change)	Lung (n-fold change)	Brain (n-fold change)	Lung (n-fold change)
PSMD2	Ssc.1642	F: tggggagaataagcgttttg R: tattcatgaccccatgatgc	Ref	Ref	Ref	Ref
AKT1	Ssc.29760	F: tgggcgacttcatccttg R: tggaagtggcagtgagca	ND^a^	1.68	ND	2.19
CDC42	Ssc.6687	F: aaagtgggtgcctgagata R: ctccacatacttgacagcc	-^b^	2.03	-	7.38
LY96	Ssc.25550	F:cattgcacgaagagacataca R: tgtattcacagtctctcccttc	1.37	3.32	6.91	9.23
PIK3R1	Ssc.49949	F: cccaggaaatccaaatga R: ggtcctcctccaaccttc	-	-	0.61	0.45
SERPINE1	Ssc.9781	F: ccagcagcagatccaaga R: cggaacagcctgaagaagt	-1.66	2.36	-0.64	4.28

^a^ND, not done;

^b^-, not changed or absent.

## Results

### Microarray analysis of gene expression profiles in brain and lung

Six brain samples and four lung samples were used for microarray hybridization and qRT-PCR, and two of the lung samples were excluded as they were found to be degraded. Table 2 shows the number of differentially expressed human probe sets initially identified in brain and lung tissues (p-value < 0.01 and p-value < 0.05). Based on BLAST analysis, those probes with putative pig gene homologues have been considered for further analysis and numbers are shown in table 2. This avoids making assumptions about other probes that detect expression changes but have weaker matches to pig ESTs. Most probes with porcine homologues remained unchanged, and few showed a reduction in transcription level by microarray analysis. For example, expression of only 4 (60-70 bp human match category) and 1 (50-59 bp human match category) were decreased in infected lung tissue (p-value < 0.01). In contrast, a large number of host transcripts were induced in response to wild type PRV infection (table 2). Here we identified 120 and 866 up-regulated transcripts in brain and lung (p-value < 0.01) with pig: human matches ≥ 60 bp, and 42 and 259 genes with matches of 50-59 bp for further gene ontology and pathway classification (table 2).

**Table 2:** Number of probe sets and pig gene homologues in brain and lung tissues affected by wild type PRV infection

p- value	Up/down regulated	Brain				Lung			
		
		A	B	C	D	A	B	C	D
p-value < 0.01	down	253	35(34)	14(14)	17	195	4(4)	1(1)	11
	up	528	132(120)	44(42)	115	2283	888(866)	261(259)	424
p-value < 0.05	down	588	77(76)	26(26)	43	1657	25(24)	4(4)	51
	up	879	209(196)	69(67)	173	3284	1122(1075)	357(355)	545

A = Total number of differentially expressed human probes.

B = Total number of pig Unigene matches of 60-70 basepairs (subset of verified gene or thologues).

C = Total number of pig Unigene matches of 50-59 basepairs (subset of verified gene or thologues).

D = Total number of EST matches >50 basepairs with no assigned Unigene ID.

Of the transcripts with matches ≥ 60 bp, 76, corresponding to 74 unique pig gene homologues, are up-regulated in common between the two tissues and are listed in Additional file 1. Forty-four probe sets corresponding to 41 unique pig gene homologues with matches of 50-59 bp also displayed increased expression in both tissues after infection by wild type PRV (Additional file 1).

### Gene Ontology and bioinformatics analysis

To characterize the sets of functionally related genes that are differentially expressed between the infected and uninfected group, we used the Onto- Express tool to classify up-regulated genes in each tissue according to their biological process. Table 3 summarizes the largest classes identified on the basis of biological process. Twelve defined biological processes with matches ≥ 60 bp, and 10 with matches of 50-59 bp, are observed in brain at least two fold more often than expected. In comparison, 9 processes with matches ≥ 60 bp, and only 4 with matches of 50-59 bp are over-represented in lung, although the total number of up-regulated genes in lung is more than that in brain tissue (table 2).

**Table 3:** Classes of biological processes involving up regulatedpig gene homologues (p-value < 0.01) in brain and lung tissues infected with wild type PRV.

Biological Process	Library	Brain Pig Unigene Matches over 60 base-pairs (gene homologues)	Brain Pig Ungene Matches between 50- 59b base-pairs (gene homologues)	Lung Pig Unigene Matches over 60 base-pairs (gene homologues)	Lung Pig Unigene Matches between 50-59b base-pairs (gene homologues)
Apoptosis	230	3*	0	30*	4
Biological function unknown	472	4	0	31	10
Cation transport	139	2*	3*	0	2
Cell adhesion	429	8*	5*	11	4
Cell cycle	303	3	0	31*	3
Cell differentiation	230	4*	1*	7	2
Immune response	255	0	0	7	3
Intracellular protein transport	135	5*	0	16*	5*
Intracellular signaling cascade	285	4*	0	14	3
Ion transport	304	5*	1	6	4
Metabolism	280	3*	3	15*	9*
Nervous system development	239	9*	3*	9	3
Protein amino acid dephosphorylation	108	1	2*	14*	1
Protein amino acid phosphorylation	412	1	2*	29	7
Protein folding	165	4*	0	27*	5*
Protein transport	217	2	2*	33*	4
Proton transport	47	1*	2*	3	7*
Regulation of progression through cell cycle	215	2	2*	20*	4
Regulation of transcription, DNA dependent	1285	9	2	73	7
Signal transduction	1110	6	1	40	6
Synaptic transmission	164	4*	4*	5	1
Transcription	945	7	1	63	7
Ubiquitin cycle	217	1	0	30*	4

* Biological processes with at least two times the expected number of genes (calculated from the library composition).

### Pathways affected by wild- type PRV infection in brain and lung

One indication that the observed transcript differences (p-value < 0.01) may have biological relevance is that sets of genes in known pathways show coordinated regulation. Accordingly, the functionally classified genes were mapped to known cellular pathways. Fifteen pathways with at least five times the expected number of genes (matches ≥ 60 bp) have been highlighted with pathway-express in the infected brain. Interestingly, most of them belong to neurodegenerative disorders, nervous system and immune system pathways. Twelve pathways (including the calcium signaling pathway, the phosphatidylinositol signaling system and the TGFβ signaling pathway) with at least five times the expected number of genes (matches of 50-59 bp) were also highlighted in the infected brain. However only 4 pathways (ubiquitin mediated proteolysis and prion disease, matches ≥ 60 bp; ALS and mTOR signaling pathway, matches of 50-59 bp) showed at least five times the expected number of genes in the infected lung (table 4). Interestingly, ubiquitination of PRV glycoproteins for vaccination has been shown to be related to decreased cellular immune responses following wild type infection. Additional file 2 lists the details of genes that were assigned to cellular processes, environmental information processes and human disease pathways.

**Table 4:** Cellular Pathways involving up-regulated (p-value < 0.01) pig gene homologues in brain and lung tissues infected with wild type PRV.

Pathway Name	Library	Brain Pig Unigene Matches over 60 base-pairs (gene homologues)	Brain Pig Unigene Matches between 50- 59b base- pairs (gene homologues)	Lung Pig Unigene Matches over 60 base-pairs (gene homologues)	Lung Pig Unigene Matches between 50-59b base-pairs (gene homologues)
***Behavior***					
Circadian rhythm	17	0	0	1	0
***Cancers***					
Colorectal cancer	73	2*	0	9	0
***Cell Communication***					
Adherens junction	72	2*	0	9	2
Focal adhesion	187	4	1	11	4
Gap junction	91	3*	0	5	0
Tight junction	106	2	0	13	3
***Cell Growth and Death***					
Apoptosis	81	0	0	3	1
Cell cycle	105	1	1*	12	4
***Cell Motility***					
Regulation of actin cytoskeleton	195	6*	0	12	2
***Development***					
Axon guidance	119	2	1	7	3
***Endocrine System***					
Adipocytokine signaling pathway	68	1	0	2	2
GnRH signaling pathway	94	3*	2*	6	1
Insulin signaling pathway	125	2	1	8	1
***Folding, Sorting and Degradation***					
Regulation of autophagy	24	0	0	2	0
SNARE interactions in vesicular transport	28	0	1*	3	1
Ubiquitin mediated proteolysis	41	0	1*	11*	1
***Immune System***					
Antigen processing and presentation	80	0	0	3	0
B cell receptor signaling pathway	61	2*	0	6	1
Complement and coagulation cascades	60	0	0	1	0
Fc epsilon RI signaling pathway	73	2*	0	4	1
Leukocyte transendothelial migration	111	1	0	6	3
Natural killer cell mediated cytotoxicity	119	2	0	4	2
T cell receptor signaling pathway	87	3*	0	5	3
Toll-like receptor signaling pathway	87	1	0	6	0
***Infectious Diseases***					
Epithelial cell signaling in Helicobacter pylori infection	45	1	0	5	1
***Metabolic Disorders***					
Type I diabetes mellitus	42	1	1*	2	0
***Nervous System***					
Long-term depression	74	2*	0	5	0
Long-term potentiation	65	2*	2*	5	3
***Neurodegenerative disorders***					
Neurodegenerative disorders	33	2*	1*	0	1
Alzheimer's disease	18	0	0	2	0
Amyotrophic lateral sclerosis (ALS)	17	3*	0	0	2*
Dentatorubropallidoluysian atrophy (DRPLA)	12	0	0	1	0
Huntington's disease	26	2*	1*	4	0
Parkinson's disease	15	1*	0	0	0
Prion disease	10	1*	0	3*	0
***Sensory System***					
Olfactory transduction	30	0	2*	2	0
Taste transduction	51	1	0	1	0
***Signal Transduction***					
Calcium signaling pathway	173	0	4*	3	3
Hedgehog signaling pathway	54	0	0	3	0
Jak-STAT signaling pathway	147	0	0	6	2
MAPK signaling pathway	267	5	2	18	7
mTOR signaling pathway	44	0	0	4	3*
Notch signaling pathway	39	0	0	1	0
Phosphatidylinositol signaling system	77	0	1*	0	0
TGF-beta signaling pathway	70	1	1*	11	0
VEGF signaling pathway	68	3	0	5	2
Wnt signaling pathway	138	0	1	12	2
***Signaling Molecules and Interaction***					
Cell adhesion molecules (CAMs)	123	2	1	3	2
Cytokine-cytokine receptor interaction	242	0	0	3	2
ECM-receptor interaction	85	1	0	3	2
Neuroactive ligand-receptor interaction	275	1	0	1	0

* Cellular pathways with at least five times the expected number of genes (calculated from the library composition).

Ten genes up-regulated in both tissues by wild-type PRV infection segregated into known pathways (Additional file 2). Most of them are involved in multiple pathways, such as *SPP1 *in the immune response pathway, the ECM-receptor interaction and focal adhesion pathway, and *FOS *and *CDC42 *in the T cell receptor signaling pathway and MAPK signaling pathway. Moreover, it is also interesting to note that a few genes such as *SERPINE1 *and *LCP2 *respond differently in the two tissues studied, and while some of the pathways responding to the infection are ubiquitous, others appear to be tissue specific (Additional file 2).

### qRT-PCR analysis for validation of microarray results

In order to verify the data obtained in the microarray experiment, we confirmed the expression profile of 5 selected genes with different patterns of expression: *LY96 *is differentially expressed in the same direction in both tissues; *SERPINE1 *is down-regulated in the brain but up-regulated in the lung after infection; *CDC42 *and *AKT1 *are significantly up-regulated in lung tissue only, and *PIK3R1 *is not significantly differentially expressed. Results from real-time quantitative RT-PCR confirmed the direction of expression (up or down-regulated) obtained by microarray analysis in the 5 genes tested (table 1). The magnitude of the fold change is not the same. This is most probably due to the fact that the array analysis is based on a cross-species hybridization whereas the RT-PCR has been performed using species homologous primers. It is likely that the RT-PCR analysis reflects more accurately the fold change in expression.

## Discussion

The virus replication cycle involves a series of host-virus interactive processes causing changes in expression of cellular genes, and an infected host activates both innate and adaptive immune responses to eliminate the invading virus [17]. The pig is an ideal animal model for studying human diseases, so the identification of pig model biomarkers for viral diseases is an important step towards identification of human counterparts. The identification of biomarkers has already been proposed as a way to create new diagnostic tools for specific microbial infection [18,19].

Previous studies have shown the value of using cross-species hybridization [20]. Here, using the Illumina human oligonucleotide Refset in a cross-species study we identified hundreds of probes with expression levels that were altered in brain and lung following wild type PRV infection of young piglets, which typically have more severe clinical manifestations than the adult. In adult pigs one observes mainly, or exclusively, the respiratory symptoms, whereas in piglets and rodent hosts there is invariably invasion of the central nervous system (CNS) [21,22]: piglets exhibit signs in the form of tremor, trembling and incoordination. Thus piglets permit the potential identification of a wider spectrum of genes involved in the disease processes in different tissues.

Classification of the genes that are differentially expressed in piglet brain into functional groups(Additional file 2) revealed that several genes are also implicated in human neurodegenerative disorders. These include genes in the pathways for amyotrophic lateral sclerosis (*NEF3, NEFL, NEFH*), Huntington's disease (*CALM3, CLTC, CLTB*), neurodegenerative disorders (*APLP1, NEFH, FBXW7*), Parkinson's disease (*GPR37*) and prion disease (*APLP1, NFE2L2*). It is not known if these transcriptional changes are primary or secondary effects of the PRV infection.

Several members of the immune response pathways (eg. the B cell receptor signaling pathway, the Fc epsilon RI signaling pathway, natural killer cell mediated cytotoxicity and the T cell receptor signaling pathway) were also transcriptionally regulated by PRV infection in brain. This is in agreement with the results from PRV or HSV-1 infection in primary cultures of rat embryonic fibroblasts [5]. In addition, similar changes to immune response pathway (e.g. antigen processing and presentation, complement and coagulation cascades), cell differentiation and metabolism pathway genes have been described in the host following PRV infection in rat CNS [6]. Our experiment not only identified pathways, but also several genes in common with these previous studies: *FOS *and *LCP2*, both involved in T cell receptor signalling pathways; the TGFβ signal transduction pathway components *ID4 *and *THBS4*, highlighted in the study of PRV infection of primary cultures of rat embryonic fibroblasts [5,6]; and *SERPINE-1*, identified in both earlier rat studies. These genes may be potential diagnostic and therapeutic targets for viral encephalitis and other neurodegenerative or neuroinflammatory diseases.

Several genes of the TGFβ pathway were also identified here in the infected lung tissue (e.g. *PPP2CA, PPP2CB, ID2, ID3 *and *ID4*). After PRV infection, most older swine exhibit signs of respiratory disease, and the study of the lung is therefore important for understanding what genes may be involved in the disease process. We identified 1130 differentially expressed probes as a result of wild-type PRV infection; this is 5 times higher than in the brain. The lung may be more transcriptionally active, or have a more pronounced immune response that might involve more immune cell types than the brain. In addition, we have identified 5 possible viral receptors, normally necessary for the spread of virus between cells, up-regulated in the infected lung: *HveC (PVRL1), PVRL3, HveD (PVR, CD155), HS3ST4 *and *HS3ST5 *[23,24]. Finally, a number of members of the TNF receptor family, usually involved in apoptosis, were identified (*TNFRSF10, 21, 25, 9, 17, 8, 1α*). This apoptotic pathway was also described in the study of HSV infection of glial cell types [25]. However, the result is interesting as the family member TNFRSF14 has been shown to be involved in some cases of viral entry, but we do not know whether these other family members are involved in viral entry and cell fusion, or only have a downstream role.

Numerous other genes involved in cellular proliferation (YWHAB, BUB1, PCNA, GADD45, MCM7, CDK4, CDK7) and apoptosis (PRKACA, PDCD8, AKT1, PPP3CA), were identified. These pathways were previously described following PRV and HSV infection in several models [5,25] and might reflect the proliferation of immune cells. A number of other genes differentially expressed in the lung, such as HSPD1, HSPB2, SERPINE-1, are in common with human and mouse models infected by HSV-1 [5,26].

Recently, Flori et al [27] have published a time course transcription profiling study (based on the Qiagen 8541 gene porcine oligonucleotide array and a 1789 porcine and PRV cDNA array) investigating both the PRV transcriptome and the host transcriptome responses of PK15 (porcine kidney) cells in culture. This study reports the early down-regulation of many cellular genes in contrast to the data in this paper. This difference most probably arises from the artificial cell culture study where there is a homogeneous cell population, whereas our present study is an in vivo investigation of complex tissues. It is entirely possible that minor tissue cell types exhibit down-regulation of many of the same genes, however, their contribution to the overall signal renders these changes undetectable. This may also explain the differences in gene expression changes for shared genes between lung and brain. In general, fold changes are lower in brain which probably reflects the complexity of cell types in the tissue, not all of which may respond equally to infection. Nevertheless, it is clear that the Flori et al. study has also observed changes in gene expression in the main categories of cellular functions described in this paper; most notably genes involved in immune responses and cell proliferation and apoptosis.

Genetic differences have been reported in the susceptibility to PRV between European Large White and Chinese Meishan pigs, with differences in cell-mediated and humoral immunity, as well as the outward clinical signs in young pigs [28]. In this study we identified several differentially expressed genes located at or close to the QTL regions previously reported. Two genes (CD36 and NPL) up-regulated in the infected brain and lung are located near the SW749 marker, which is associated with changes in body temperature and neurological signs. ETA1 (alias SPP1), which is involved in the recruitment of T-lymphocytes [29,30], was up-regulated in both tissues after natural PRV infection, and is linked to the QTL region of chromosome 8. One of the PRV receptors, PVRL3, which is differentially expressed in infected lung, is linked to a QTL on chromosome 13. CLDN7, which is involved with cell communication, was down-regulated in the infected brain and is linked to a QTL on chromosome 13 associated with neurological signs.

## Conclusion

By combining the array data presented here with the information from the previous QTL study, it may be possible to identify the best candidates for the clinical features and increased resistance to PRV infection. In addition, further studies and functional analysis of these candidates will broaden the scientific understanding of PRV infection, provide biomarkers to use as diagnostic tools, and may also lead to the development of novel antiviral treatments and/or the application of marker assisted selection for disease resistance.

## Abbreviations

μL: microliter(s); AKT1: v-akt murine thymoma viral oncogene homolog 1; ALS: amyotrophic lateral sclerosis; APLP1: amyloid beta (A4) precursor-like protein 1; BHV-1: bovine herpesvirus 1; bp: basepair(s); BUB1: budding uninhibited by benzimidazoles 1; CALM3: calmodulin 3; CDC42: cell division cycle 42; CDK4: cyclin-dependent kinase 4; CDK7: cyclin-dependent kinase 7; cDNA: complementary deoxyribonucleic acid; CLTB: clathrin light chain; CLTC: clathrin heavy chain; CNS: central nervous system; Ct: threshold cycle; DNA: deoxyribonucleic acid; EST: expressed sequence tag; FBXW7: F-box and WD repeat domain containing 7; FOS: v-fos FBJ murine osteosarcoma viral oncogene homolog; GADD45: growth arrest and DNA-damage-inducible alpha; GPR37: G protein-coupled receptor 37; h: hour; HS3ST4: heparan sulfate (glucosamine) 3-O-sulfotransferase 5; HS3ST5: heparan sulfate (glucosamine) 3-O-sulfotransferase 5; HSPB2: heat shock 27 kDa protein 2; HSPD1: heat shock 60 kDa protein 1; HveC (PVRL1): herpesvirus entry mediator C (poliovirus receptor-related 1); HveD (PVR): herpesvirus entry mediator D (poliovirus receptor); ID2: inhibitor of DNA binding 2; ID3: inhibitor of DNA binding 3; ID4: inhibitor of DNA binding 4; LCP2: lymphocyte cytosolic protein 2; MAPK: mitogen-activated protein kinase; MCM7: minichromosome maintenance complex component 7; mRNA: messenger ribonucleic acid; mTOR: mechanistic target of rapamycin; Na: sodium; NEF3: neurofilament, medium polypeptide; NEFH: neurofilament, heavy polypeptide; NEFL: neurofilament, light polypeptide; NFE2L2: nuclear factor (erythroid-derived 2)-like 2; ng: nanogram(s); PCNA: proliferating cell nuclear antigen; PCR: polymerase chain reaction; PDCD8: programmed cell death 8; PIK3R1: phosphoinositide-3-kinase regulatory subunit 1; PPP2CA: protein phosphatase 2 catalytic subunit alpha isoform; PPP2CB: protein phosphatase 2 catalytic subunit beta isoform; PPP3CA: protein phosphatase 3 catalytic subunit alpha isoform; PRKACA: protein kinase, cAMP-dependent, catalytic, alpha; PRRS: porcine reproductive and respiratory syndrome; PRV: pseudorabies virus; PSMD2: 26S proteasome non-ATPase regulatory subunit 2; PVRL3: poliovirus receptor-related 3; qRT: quantitative real time; QTL: quantitative trait locus; rRNA: ribosomal ribonucleic acid; SERPINE1: plasminogen activator inhibitor, type I; SPP1: secreted phosphoprotein 1; TGFβ: transforming growth factor, beta; THBS4: thrombospondin 4; TNFRSF: tumor necrosis factor receptor superfamily; tRNA: transfer ribonucleic acid; YWHAB: tyrosine 3-monooxygenase/tryptophan 5-monooxygenase activation protein, beta polypeptide.

## Authors' contributions

JFY, SJZ and OJ performed the microarray experiments. RAF and OEC contributed towards the data analysis. GHZ carried out animal experiments and sample collection. CAS and NAA contributed intellectually to the study, and to manuscript preparation. All authors have read and approved the final manuscript.

## Supplementary Material

Additional file 1 **Pig gene homologues up-regulated in both tissues (brain and lung) by wild type PRV infection**. The data provided represent the Pig gene homologues up-regulated in both tissues (brain and lung) by wild type PRV infection Click here for file

Additional file 2 **Pathways of pig gene homologues regulated in brain and lung tissues by wild type PRV infection**. Data represents the pathways of pig gene homologues regulated in brain and lung tissues by wild type PRV infection. Click here for file
