# Supplementary material for: Global transcriptional response of pig brain and lung to natural infection by Pseudorabies virus
Source: BMC Microbiol. 2009 Dec 1;9:246. doi: 10.1186/1471-2180-9-246 (PMC2793263; doi:10.1186/1471-2180-9-246)
Supplement: Additional file 1 — Pig gene homologues up-regulated in both tissues (brain and lung) by wild type PRV infection. The data provided represent the Pig gene homologues up-regulated in both tissues (brain and lung) by wild type PRV infection [file 1471-2180-9-246-S1.DOC]

**Supplementary Table 1.** Pig gene homologues up-regulated in both tissues (brain and lung) by wild type PRV infection

| No. of probe set | **Pig Unigene id** | **Unigene Symbol** | **Number of Match Bases** | Fold Change | |
| --- | --- | --- | --- | --- | --- |
|  |  |  |  | Brain | Lung |
| HG_010_00042 | Ssc.3708 | NDUFA12 | 66 | 1.67 | 6.04 |
| HG_010_00424 | Ssc.1228 | MARCKS | 70 | 1.66 | 3.57 |
| HG_010_00634 | Ssc.47819 | LIN7B | 63 | 1.85 | 2.06 |
| HG_010_00799 | Ssc.19235 | TMEFF2 | 68 | 1.49 | 2.47 |
| HG_010_00812 | Ssc.26717 | ARMCX5 | 66 | 1.47 | 2.41 |
| HG_010_01160 | Ssc.27281 | ARID4A | 63 | 1.60 | 2.13 |
| HG_010_01407 | Ssc.47887 | ASAP | 62 | 1.68 | 3.18 |
| HG_010_01709 | Ssc.16336 | ME1 | 63 | 1.83 | 1.73 |
| HG_010_01827 | Ssc.26469 | LRP16 | 68 | 1.54 | 1.85 |
| HG_010_02050 | Ssc.1837 | Sep7 | 65 | 1.84 | 4.48 |
| HG_010_02533 | Ssc.17758 | TMEM147 | 66 | 1.51 | 3.36 |
| HG_010_02783 | Ssc.8835 | MAP2K1 | 68 | 1.69 | 1.97 |
| HG_010_03884 | Ssc.8274 | PFN2 | 70 | 2.43 | 3.68 |
| HG_010_03886 | Ssc.23782 | PURA | 61 | 1.50 | 2.24 |
| HG_010_04076 | Ssc.4389 | FKBP1B | 62 | 2.20 | 1.62 |
| HG_010_04079 | Ssc.7555 | FKBP3 | 67 | 1.76 | 3.78 |
| HG_010_04083 | Ssc.16869 | FKBP8 | 60 | 2.22 | 2.31 |
| HG_010_04654 | Ssc.45584 | TUBA1 | 66 | 2.22 | 3.03 |
| HG_010_04727 | Ssc.8282 | Sep4 | 67 | 2.37 | 3.24 |
| HG_010_04730 | Ssc.8282 | Sep4 | 66 | 2.13 | 3.04 |
| HG_010_04789 | Ssc.12256 | CCDC104 | 63 | 1.55 | 2.28 |
| HG_010_04851 | Ssc.8250 | KIFAP3 | 63 | 1.61 | 1.93 |
| HG_010_05005 | Ssc.43923 | SORL1 | 66 | 1.73 | 3.32 |
| HG_010_05036 | Ssc.3503 | SIRPA | 62 | 1.62 | 1.62 |
| HG_010_06866 | Ssc.34434 | OPA1 | 61 | 1.61 | 3.10 |
| HG_010_07208 | Ssc.9645 | ID4 | 70 | 1.75 | 3.21 |
| HG_010_07570 | Ssc.15584 | C12orf57 | 62 | 1.58 | 2.36 |
| HG_010_07577 | Ssc.1585 | GPRASP2 | 61 | 1.82 | 2.67 |
| HG_010_07679 | Ssc.7201 | NDUFS2 | 65 | 1.49 | 3.50 |
| HG_010_07757 | Ssc.25129 | MID1IP1 | 68 | 1.83 | 3.65 |
| HG_010_07783 | Ssc.9048 | CXXC5 | 61 | 1.80 | 2.76 |
| HG_010_07867 | Ssc.9244 | NCKAP1 | 61 | 1.56 | 2.60 |
| HG_010_08527 | Ssc.26315 | LOC196394 | 64 | 1.48 | 2.97 |
| HG_010_09880 | Ssc.6447 | CAMK2N1 | 65 | 2.54 | 2.36 |
| HG_010_10015 | Ssc.12091 | SMARCA1 | 69 | 1.50 | 2.42 |
| HG_010_10363 | Ssc.3480 | YWHAB | 60 | 1.47 | 3.16 |
| HG_010_10399 | Ssc.6531 | SPARCL1 | 66 | 1.75 | 3.08 |
| HG_010_10415 | Ssc.5842 | KLHL2 | 67 | 1.54 | 2.11 |
| HG_010_10624 | Ssc.20646 | RAB30 | 65 | 2.01 | 2.87 |
| HG_010_10640 | Ssc.295 | GLS | 62 | 1.50 | 2.31 |
| HG_010_11334 | Ssc.44898 | ACYP1 | 62 | 1.55 | 3.14 |
| HG_010_11390 | Ssc.6980 | BCL7A | 64 | 1.92 | 2.34 |
| HG_010_11699 | Ssc.11103 | MDH1 | 62 | 1.79 | 2.77 |
| HG_010_13200 | Ssc.915 | C14orf4 | 70 | 1.89 | 4.01 |
| HG_010_13265 | Ssc.26724 | PPP1R9A | 62 | 1.95 | 2.47 |
| HG_010_13609 | Ssc.19345 | SFRS14 | 61 | 1.63 | 1.71 |
| HG_010_14981 | Ssc.1441 | DCTN3 | 68 | 1.85 | 2.34 |
| HG_010_15617 | Ssc.6729 | ATP5A1 | 66 | 1.47 | 3.04 |
| HG_010_16592 | Ssc.4961 | EDF1 | 63 | 1.53 | 2.62 |
| HG_010_16593 | Ssc.4961 | EDF1 | 63 | 1.88 | 3.75 |
| HG_010_16949 | Ssc.5464 | CPE | 61 | 2.65 | 3.24 |
| HG_010_17177 | Ssc.24290 | BLOC1S1 | 64 | 1.64 | 2.21 |
| HG_010_17531 | Ssc.2312 | NDUFS8 | 62 | 1.58 | 3.20 |
| HG_010_17795 | Ssc.6106 | PTPRD | 66 | 2.05 | 1.87 |
| HG_010_17858 | Ssc.25999 | RTCD1 | 65 | 1.49 | 3.10 |
| HG_010_17930 | Ssc.45258 | SLC12A2 | 64 | 1.47 | 2.64 |
| HG_010_17963 | Ssc.790 | SNRPB | 60 | 1.54 | 2.98 |
| HG_010_18032 | Ssc.49904 | TCF4 | 67 | 1.48 | 2.45 |
| HG_010_18144 | Ssc.5790 | IHPK2 | 66 | 1.59 | 3.49 |
| HG_010_18160 | Ssc.14072 | WASF1 | 68 | 2.35 | 2.36 |
| HG_010_18446 | Ssc.19546 | NME1 | 63 | 1.62 | 3.62 |
| HG_010_18538 | Ssc.13397 | ARL3 | 67 | 1.79 | 2.54 |
| HG_010_18594 | Ssc.7584 | CLTC | 68 | 1.99 | 5.77 |
| HG_010_18674 | Ssc.45658 | ETV5 | 67 | 1.72 | 2.80 |
| HG_010_18889 | Ssc.101 | SPP1 | 62 | 1.75 | 4.85 |
| HG_010_18984 | Ssc.47325 | LGI1 | 69 | 1.70 | 1.63 |
| HG_010_19624 | Ssc.979 | S100A1 | 65 | 2.24 | 3.66 |
| HG_010_19775 | Ssc.6305 | KHDRBS3 | 65 | 1.92 | 1.67 |
| HG_010_20028 | Ssc.7163 | CLTB | 63 | 1.62 | 2.58 |
| HG_010_20158 | Ssc.1555 | FOS | 67 | 1.89 | 2.63 |
| HG_010_20256 | Ssc.10679 | KIF3A | 65 | 1.66 | 1.86 |
| HG_010_20333 | Ssc.47776 | MMD | 63 | 1.52 | 2.38 |
| HG_010_20511 | Ssc.9714 | LMO4 | 66 | 1.99 | 3.80 |
| HG_010_20768 | Ssc.6666 | SCHIP1 | 67 | 1.79 | 2.48 |
| HG_010_21606 | Ssc.22615 | GTF2A1 | 70 | 1.68 | 3.28 |
| HG_010_22222 | Ssc.18226 | AURKAIP1 | 61 | 1.80 | 2.32 |
| HG_010_01051 | Ssc.6373 | NOC4L | 53 | 1.37 | 1.64 |
| HG_010_01650 | Ssc.8883 | NDFIP1 | 58 | 1.59 | 3.13 |
| HG_010_02100 | Ssc.44854 | STARD3NL | 58 | 1.37 | 1.73 |
| HG_010_02480 | Ssc.24271 | DYNLRB1 | 58 | 1.69 | 1.83 |
| HG_010_03241 | Ssc.13002 | YIF1B | 51 | 1.43 | 2.41 |
| HG_010_03245 | Ssc.5504 | TRIM37 | 52 | 1.45 | 1.47 |
| HG_010_03567 | Ssc.2250 | FBXW7 | 58 | 2.13 | 2.18 |
| HG_010_03825 | Ssc.5035 | MRPS18A | 54 | 1.48 | 2.32 |
| HG_010_04028 | Ssc.9742 | COX5A | 57 | 1.71 | 3.22 |
| HG_010_04055 | Ssc.48950 | FEZ1 | 57 | 2.33 | 2.15 |
| HG_010_04066 | Ssc.4474 | ATP6AP1 | 56 | 1.48 | 1.91 |
| HG_010_04626 | Ssc.16429 | CCNI | 52 | 1.49 | 3.73 |
| HG_010_04751 | Ssc.16681 | COX6A1 | 55 | 1.34 | 4.88 |
| HG_010_06844 | Ssc.8049 | SNAP25 | 57 | 3.38 | 2.01 |
| HG_010_08008 | Ssc.15645 | TCEA2 | 59 | 1.56 | 1.40 |
| HG_010_10366 | Ssc.3480 | YWHAB | 53 | 1.56 | 3.22 |
| HG_010_10536 | Ssc.10720 | ARMCX2 | 55 | 1.46 | 1.50 |
| HG_010_10651 | Ssc.22623 | PEG3 | 51 | 1.89 | 2.17 |
| HG_010_10742 | Ssc.2133 | NAV1 | 59 | 1.45 | 2.17 |
| HG_010_12553 | Ssc.11151 | CCDC66 | 54 | 1.36 | 2.32 |
| HG_010_15483 | Ssc.9752 | SNX3 | 58 | 1.36 | 2.74 |
| HG_010_16639 | Ssc.10380 | ATP2A2 | 58 | 1.50 | 2.41 |
| HG_010_16786 | Ssc.10380 | ATP2A2 | 52 | 1.87 | 1.80 |
| HG_010_16787 | Ssc.7985 | ATP2B1 | 55 | 1.81 | 2.57 |
| HG_010_16791 | Ssc.17183 | ATP5D | 51 | 1.45 | 1.71 |
| HG_010_17208 | Ssc.3528 | GOT1 | 55 | 1.65 | 2.10 |
| HG_010_17274 | Ssc.5615 | HK1 | 57 | 1.52 | 2.62 |
| HG_010_18734 | Ssc.16670 | IDH3G | 59 | 1.38 | 3.18 |
| HG_010_18785 | Ssc.1311 | NDUFB2 | 51 | 1.47 | 2.76 |
| HG_010_19034 | Ssc.44921 | SLC4A3 | 58 | 1.72 | 1.41 |
| HG_010_19489 | Ssc.14361 | OLFM1 | 59 | 2.08 | 2.44 |
| HG_010_19649 | Ssc.7629 | TIMM23 | 59 | 1.33 | 3.42 |
| HG_010_19685 | Ssc.13592 | ZMYND11 | 57 | 1.40 | 2.80 |
| HG_010_19829 | Ssc.26271 | EBNA1BP2 | 52 | 1.59 | 2.52 |
| HG_010_20109 | Ssc.22475 | NTHL1 | 56 | 1.38 | 1.42 |
| HG_010_20759 | Ssc.17272 | UQCRQ | 54 | 1.34 | 3.41 |
| HG_010_20840 | Ssc.11211 | C11orf51 | 52 | 1.43 | 3.03 |
| HG_010_20864 | Ssc.3480 | YWHAB | 56 | 1.70 | 3.93 |
| HG_010_20865 | Ssc.19964 | HIRIP5 | 58 | 1.37 | 4.57 |
| HG_010_21431 | Ssc.23192 | DCXR | 53 | 1.40 | 1.98 |
| HG_010_21481 | Ssc.46992 | REEP2 | 59 | 1.71 | 1.42 |
| HG_010_21482 | Ssc.5616 | ARMCX1 | 59 | 1.68 | 1.96 |
| HG_010_22599 | Ssc.13656 | SMYD2 | 56 | 1.63 | 3.25 |
| HG_010_22656 | Ssc.16624 | CA11 | 58 | 2.10 | 1.74 |
