# Supplementary material for: Global transcriptional response of pig brain and lung to natural infection by Pseudorabies virus
Source: BMC Microbiol. 2009 Dec 1;9:246. doi: 10.1186/1471-2180-9-246 (PMC2793263; doi:10.1186/1471-2180-9-246)
Supplement: Additional file 2 — Pathways of pig gene homologues regulated in brain and lung tissues by wild type PRV infection. Data represents the pathways of pig gene homologues regulated in brain and lung tissues by wild type PRV infection. [file 1471-2180-9-246-S2.DOC]

**Supplementary Table 2 Pathways of pig gene homologues regulated in brain and lung tissues** by wild type PRV infection

| **Gene Symbol** | **Unigene id** | | | **Brain Pig Unigene Matches over 60 base-pairs (gene homologues)** | **Brain Pig Ungene Matches between 50-59b base-pairs (gene homologues)** | **Lung Pig Unigene Matches over 60 base-pairs (gene homologues)** | **Lung Pig Unigene Matches between 50-59b base-pairs (gene homologues)** |
| --- | --- | --- | --- | --- | --- | --- | --- |
| **Behavior** | | | |  |  |  |  |
| ***Circadian rhythm*** | | | |  |  |  |  |
| *ARNTL* | Ssc.25227 | | | |  | 2.55 |  |
| **Cancers** |  | | | |  |  |  |
| ***Colorectal cancer*** | | | |  |  |  |  |
| *RAC1* | Ssc.7247 | | |  |  | 3.44 |  |
| *APC* | Ssc.11053 | | | |  | 3.12 |  |
| *CTNNB1* | Ssc.14003 | | | |  | 2.76 |  |
| *FOS* | Ssc.1555 | | | 1.89 |  | 2.63 |  |
| *MAP2K1* | Ssc.8835 | | | 1.69 |  | 1.97 |  |
| *GSK3B* | Ssc.14176 |  | | | | 1.96 |  |
| *MSH6* | Ssc.24063 |  | | | | 1.85 |  |
| *MYC* | Ssc.21433 |  | | | | 1.83 |  |
| *AKT1* | Ssc.29760 |  | | | | 1.68 |  |
| **Cell Communication** | | | | |  |  |  |
| ***Adherens junction*** | | | |  |  |  |  |
| *RAC1* | Ssc.7247 | | |  |  | 3.44 |  |
| *PVRL3* | Ssc.27518 | | | |  | 2.96 |  |
| *CTNNB1* | Ssc.14003 | | | |  | 2.76 |  |
| *WASF1* | Ssc.14072 | | | 2.35 |  | 2.36 |  |
| *TJP1* | Ssc.2425 | | |  |  | 2.33 |  |
| *PTPN6* | Ssc.5530 | | |  |  | 2.10* |  |
| *CDC42* | Ssc.6687 | | | 1.69 |  | 2.03* | 2.01* |
| *LMO7* | Ssc.22793 | | | |  | 1.94 |  |
| *ACP1* | Ssc.20358 | | | |  | 1.88 |  |
| *CSNK2A2* | Ssc.4909 | | |  |  |  | 1.645 |
| ***Focal adhesion*** | | | |  |  |  |  |
| *SPP1* | Ssc.101 | | | 1.75 |  | 4.85 |  |
| *RAC1* | Ssc.7247 | | |  |  | 3.44 |  |
| *PPP1CC* | Ssc.11661 | | | |  | 3.23 |  |
| *CTNNB1* | Ssc.14003 | | | |  | 2.76 |  |
| *CDC42* | Ssc.6687 | | | 1.69 |  | 2.03 | 2.01 |
| *PDPK1* | Ssc.47335 | | | |  | 1.99 |  |
| *MAP2K1* | Ssc.8835 | | | 1.69 |  | 1.97 |  |
| *GSK3B* | Ssc.14176 | | | |  | 1.96 |  |
| *LAMB1* | Ssc.5299 | | |  |  | 1.91 |  |
| *ITGB1* | Ssc.4621 | | |  |  | 1.82 |  |
| *AKT1* | Ssc.29760 | | | |  | 1.68 |  |
| *LAMA2* | Ssc.11623 | | | |  |  | 1.88 |
| *RAP1A* | Ssc.11026 | | | |  |  | 2.52 |
| *ILK* | Ssc.2578 | | |  |  |  | 1.97 |
| *HRAS* | Ssc.16352 | | | 1.55 |  |  |  |
| *COL5A2* | Ssc.17300 | | | -1.59 |  |  |  |
| *THBS4* | Ssc.1411 | | |  | 1.50 |  |  |
| ***Gap junction*** | | | |  |  |  |  |
| *TUBA1* | Ssc.45584 | | | |  | 3.03 |  |
| *PRKACA* | Ssc.13872 | | | |  | 2.42 |  |
| *TJP1* | Ssc.2425 | | |  |  | 2.33 |  |
| *MAP2K1* | Ssc.8835 | | | 1.69 |  | 1.97 |  |
| *GNAI2* | Ssc.841 | | |  |  | 1.64 |  |
| *TUBA1* | Ssc.45584 | | | 2.22 |  |  |  |
| *HRAS* | Ssc.16352 | | | 1.55 |  |  |  |
| *CDC2* | Ssc.873 | | | -1.62* |  |  |  |
| ***Tight junction*** | | | |  |  |  |  |
| *PPP2CA* | Ssc.13983 | | | |  | 3.44 |  |
| *PPP2CB* | Ssc.236 | | |  |  | 3.39 |  |
| *CTNNB1* | Ssc.14003 | | | |  | 2.76 |  |
| *VAPA* | Ssc.17275 | | | |  | 2.72 |  |
| *TJP1* | Ssc.2425 | | |  |  | 2.33 |  |
| *CDC42* | Ssc.6687 | | | 1.69 |  | 2.03* | 2.01* |
| *CLDN7* | Ssc.825 | | | -1.49 |  | 1.93 |  |
| *PRKCH* | Ssc.2011 | | |  |  | 1.90 |  |
| *AKT1* | Ssc.29760 | | | |  | 1.68 |  |
| *CDK4* | Ssc.11051 | | | |  | 1.66 |  |
| *PPP2R2A* | Ssc.104 | | |  |  | 1.64 |  |
| *GNAI2* | Ssc.841 | | |  |  | 1.64 |  |
| *CLDN10* | Ssc.44153 | | | |  |  | 2.48 |
| *CSNK2A2* | Ssc.4909 | | |  |  |  | 1.64 |
| *HRAS* | Ssc.16352 | | | 1.55 |  |  |  |
| **Cell Growth and Death** | | | | |  |  |  |
| ***Apoptosis*** | | | |  |  |  |  |
| *PRKACA* | Ssc.13872 | | | |  | 2.42 |  |
| *PDCD8* | Ssc.19966 | | | |  | 2.00* |  |
| *AKT1* | Ssc.29760 | | | |  | 1.68 |  |
| *PPP3CA* | Ssc.15878 | | | |  |  | 1.92 |
| ***Cell cycle*** | | | |  |  |  |  |
| *YWHAB* | Ssc.3480 | | | 1.56* | 1.70* | 3.22* | 3.93* |
| *RBX1* | Ssc.20969 | | | |  | 3.30 |  |
| *ANAPC10* | Ssc.38714 | | | |  | 3.01 |  |
| *CCNH* | Ssc.28546 | | | |  | 2.85 |  |
| *HDAC2* | Ssc.3040 | | |  |  | 2.73 |  |
| *CDK7* | Ssc.13186 | | | |  | 2.65 |  |
| *CCNB1* | Ssc.9547 | | | -1.50 |  | 2.53* |  |
| *CDC16* | Ssc.21956 | | | |  | 2.13 |  |
| *GSK3B* | Ssc.14176 | | | |  | 1.96 |  |
| *PCNA* | Ssc.24287 | | -1.49 | |  | 1.76 |  |
| *ANAPC2* | Ssc.1633 | | |  |  | 1.66 |  |
| *CDK4* | Ssc.11051 | | | |  | 1.66 |  |
| *ATR* | Ssc.7780 | | |  |  |  | 2.58 |
| *MCM7* | Ssc.17030 | | | |  |  | 1.70 |
| *GADD45A* | Ssc.20913 | | | |  |  | 2.81 |
| *CDC2* | Ssc.873 | | | -1.58* |  |  |  |
| *BUB1* | Ssc.7195 | | | -1.54 |  |  |  |
| *CCNB1* | Ssc.14243 | | | | -1.50 |  |  |
| **Cell Motility** | | | |  |  |  |  |
| ***Regulation of actin cytoskeleton*** | | | | | |  |  |
| *PFN2* | Ssc.8274 | | | 2.43* |  | 3.68 |  |
| *RAC1* | Ssc.7247 | | |  |  | 3.44 |  |
| *PPP1CC* | Ssc.11661 | | | |  | 3.23 |  |
| *APC* | Ssc.11053 | | | |  | 3.12 |  |
| *NCKAP1* | Ssc.9244 | | | 1.56 |  | 2.60 |  |
| *WASF1* | Ssc.14072 | | | 2.35 |  | 2.36 |  |
| *CSK* | Ssc.44647 | | | |  | 2.29 |  |
| *CDC42* | Ssc.6687 | | | 1.69 |  | 2.03* | 2.01* |
| *MAP2K1* | Ssc.8835 | | | 1.69 |  | 1.97 |  |
| *PFN1* | Ssc.835 | | |  |  | 1.88 |  |
| *ITGB1* | Ssc.4621 | | |  |  | 1.82 |  |
| *GNG12* | Ssc.41291 | | | |  | 1.78 |  |
| *CFL2* | Ssc.7645 | | |  |  |  | 2.58 |
| *HRAS* | Ssc.16352 | | | 1.55 |  |  |  |
| *ATG3* | Ssc.7402 | | |  |  | 2.61 |  |
| *GABARAP* | Ssc.11120 | | | |  | 1.98 |  |
| **Development** | | | |  |  |  |  |
| ***Axon guidance*** | | | |  |  |  |  |
| *RAC1* | Ssc.7247 | | |  |  | 3.44 |  |
| *NTN1* | Ssc.24727 | | | |  | 2.26 |  |
| *PLXNC1* | Ssc.3879 | | |  |  | 2.04 |  |
| *CDC42* | Ssc.6687 | | | 1.69 |  | 2.03 | 2.01 |
| *GSK3B* | Ssc.14176 | | | |  | 1.98 |  |
| *ITGB1* | Ssc.4621 | | |  |  | 1.82 |  |
| *GNAI2* | Ssc.841 | | |  |  | 1.64 |  |
| *CFL2* | Ssc.7645 | | |  |  |  | 2.58 |
| *PPP3CA* | Ssc.15878 | | | |  |  | 1.92 |
| *HRAS* | Ssc.16352 | | | 1.55 |  |  |  |
| *EPHA4* | Ssc.21909 | | | | 1.79 |  |  |
| **Endocrine System** | | | |  |  |  |  |
| ***Adipocytokine signaling pathway*** | | | | | |  |  |
| *ADIPOR1* | Ssc.13269 | | | |  | 2.68 |  |
| *AKT1* | Ssc.29760 | | | |  | 1.68 |  |
| *CD36* | Ssc.37254 | | | | -1.57 |  | 1.91 |
| *JAK1* | Ssc.328 | | |  |  |  | 2.24 |
| *ACSL6* | Ssc.11255 | | | 1.66 |  |  |  |
| ***GnRH signaling pathway*** | | | | |  |  |  |
| *CAMK2D* | Ssc.14480 | | | |  | 2.73 |  |
| *PRKACA* | Ssc.13872 | | | |  | 2.42 |  |
| *MAP3K3* | Ssc.18489 | | | |  | 2.18 |  |
| *CDC42* | Ssc.6687 | | | 1.69 |  | 2.04* | 2.01* |
| *MAP2K1* | Ssc.8835 | | | 1.69 |  | 1.97 |  |
| *MAP3K4* | Ssc.4096 | | |  |  | 1.77 |  |
| *HRAS* | Ssc.16352 | | | 1.55 |  |  |  |
| *CALM3* | Ssc.3675 | | |  | 1.71 |  |  |
| ***Insulin signaling pathway*** | | | | |  |  |  |
| *RHOQ* | Ssc.9000 | | |  |  | 3.61 |  |
| *PPP1CC* | Ssc.11661 | | | |  | 3.23 |  |
| *PYGL* | Ssc.1104 | | |  |  | 2.91 |  |
| *PRKACA* | Ssc.13872 | | | |  | 2.4 |  |
| *PDPK1* | Ssc.47335 | | | |  | 1.99 |  |
| *MAP2K1* | Ssc.8835 | | | 1.69 |  | 1.97 |  |
| *GSK3B* | Ssc.14176 | | | |  | 1.96 |  |
| *AKT1* | Ssc.29760 | | | |  | 1.68 |  |
| *EIF4E2* | Ssc.18203 | | | |  |  | 1.73 |
| *HRAS* | Ssc.16352 | | | 1.55 |  |  |  |
| *CALM3* | Ssc.3675 | | |  | 1.71 |  |  |
| **Folding, Sorting and Degradation** | | | | | |  |  |
| ***SNARE interactions in vesicular transport*** | | | | | | |  |
| *VAMP4* | Ssc.28051 | | | |  | 4.23 |  |
| *GOSR2* | Ssc.44962 | | | |  | 3.32 |  |
| *BET1* | Ssc.14022 | | | |  | 2.09 |  |
| *SNAP25* | Ssc.8049 | | |  | 3.38 |  | 2.01 |
| ***Ubiquitin mediated proteolysis*** | | | | | |  |  |
| *WWP1* | Ssc.1680 | | |  |  | 3.30 |  |
| *RBX1* | Ssc.20969 | | | |  | 3.30 |  |
| *ANAPC10* | Ssc.38714 | | | |  | 3.01 |  |
| *EDD1* | Ssc.13158 | | | -1.51 |  | 2.89 |  |
| *UBE2D2* | Ssc.49951 | | | |  | 2.82 |  |
| *UBE2D3* | Ssc.7870 | | |  |  | 2.79 |  |
| *CUL2* | Ssc.9005 | | |  |  | 2.69 |  |
| *TCEB1* | Ssc.3883 | | |  |  | 2.54 |  |
| *UBE2E3* | Ssc.11673 | | | |  | 2.40 |  |
| *CDC16* | Ssc.21956 | | | |  | 2.13 |  |
| *ANAPC2* | Ssc.1633 | | |  |  | 1.66 |  |
| *FBXW7* | Ssc.2250 | | |  | 2.13 |  | 2.18 |
| **Immune System** | | | |  |  |  |  |
| ***Antigen processing and presentation*** | | | | | |  |  |
| *PDIA3* | Ssc.10997 | | | |  | 3.34 |  |
| *NFYB* | Ssc.10211 | | | |  | 2.21 |  |
| *CREB1* | Ssc.16173 | | | |  | 1.88 |  |
| ***B cell receptor signaling pathway*** | | | | | |  |  |
| *RAC1* | Ssc.7247 | | |  |  | 3.44 |  |
| *FOS* | Ssc.1555 | | | 1.89 |  | 2.63 |  |
| *BLNK* | Ssc.8594 | | |  |  | 2.47 |  |
| *PTPN6* | Ssc.5530 | | |  |  | 2.10*. |  |
| *GSK3B* | Ssc.14176 | | | |  | 1.96 |  |
| *AKT1* | Ssc.29760 | | | |  | 1.68 |  |
| *PPP3CA* | Ssc.15878 | | | |  |  | 1.92 |
| *HRAS* | Ssc.16352 | | | 1.55 |  |  |  |
| ***Complement and coagulation cascades*** | | | | | |  |  |
| *SERPINE1* | Ssc.9781 | | | -1.66 |  | 2.36 |  |
| ***Fc epsilon RI signaling pathway*** | | | | | |  |  |
| *RAC1* | Ssc.7247 | | |  |  | 3.44 |  |
| *FCER1G* | Ssc.508 | | |  |  | 3.31 |  |
| *MAP2K1* | Ssc.8835 | | | 1.69 |  | 1.97 |  |
| *AKT1* | Ssc.29760 | | | |  | 1.68 |  |
| *LCP2* | Ssc.15565 | | | | -1.54 |  | 3.69 |
| *HRAS* | Ssc.16352 | | | 1.55 |  |  |  |
| ***Leukocyte transendothelial migration*** | | | | | | |  |
| *RAC1* | Ssc.7247 | | |  |  | 3.44 |  |
| *CTNNB1* | Ssc.14003 | | | |  | 2.76 |  |
| *CDC42* | Ssc.6687 | | | 1.69 |  | 2.03*. | 2.01* |
| *CLDN7* | Ssc.825 | | | -1.49 |  | 1.93 |  |
| *ITGB1* | Ssc.4621 | | |  |  | 1.82 |  |
| *GNAI2* | Ssc.841 | | |  |  | 1.64 |  |
| *CLDN10* | Ssc.44153 | | | |  |  | 2.48 |
| *RAP1A* | Ssc.11026 | | | |  |  | 2.52 |
| ***Natural killer cell mediated cytotoxicity*** | | | | | | |  |
| *RAC1* | Ssc.7247 | | |  |  | 3.44 |  |
| *FCER1G* | Ssc.508 | | |  |  | 3.31 |  |
| *PTPN6* | Ssc.5530 | | |  |  | 2.10* |  |
| *MAP2K1* | Ssc.8835 | | | 1.69 |  | 1.97 |  |
| *LCP2* | Ssc.15565 | |  | | -1.54 |  | 3.69 |
| *PPP3CA* | Ssc.15878 | |  | |  |  | 1.92 |
| *HRAS* | Ssc.16352 | | | 1.55 |  |  |  |
| ***T cell receptor signaling pathway*** | | | | | |  |  |
| *FOS* | Ssc.1555 | | | 1.89 |  | 2.63 |  |
| *PTPN6* | Ssc.5530 | | |  |  | 2.10* |  |
| *CDC42* | Ssc.6687 | | | 1.69 |  | 2.03 | 2.01 |
| *AKT1* | Ssc.29760 | | | |  | 1.7 |  |
| *CDK4* | Ssc.11051 | | | |  | 1.6 |  |
| *LCP2* | Ssc.15565 | | | | -1.54 |  | 3.69 |
| *PPP3CA* | Ssc.15878 | | | |  |  | 1.92 |
| *HRAS* | Ssc.16352 | | | 1.55 |  |  |  |
| ***Toll-like receptor signaling pathway*** | | | | | | |  |
| *RAC1* | Ssc.7247 | | |  |  | 3.44 |  |
| *STAT1* | Ssc.6025 | | |  |  | 2.92 |  |
| *FOS* | Ssc.1555 | | | 1.89 |  | 2.63 |  |
| *MAP3K7IP2* | Ssc.34309 | | | |  | 2.08 |  |
| *AKT1* | Ssc.29760 | |  | |  | 1.68 |  |
| **Nervous System** |  | |  | |  |  |  |
| ***Long-term depression*** | | | | |  |  |  |
| *PPP2CA* | Ssc.13983 | | | |  | 3.44 |  |
| *PPP2CB* | Ssc.236 | | |  |  | 3.39 |  |
| *MAP2K1* | Ssc.8835 | | | 1.69 |  | 1.97 |  |
| *PPP2R2A* | Ssc.104 | | |  |  | 1.64 |  |
| *GNAI2* | Ssc.841 | | |  |  | 1.64 |  |
| *HRAS* | Ssc.16352 | | | 1.55 |  |  |  |
| ***Long-term potentiation*** | | | |  |  |  |  |
| *PPP1CC* | Ssc.11661 | | |  |  | 3.23 |  |
| *CAMK2D* | Ssc.14480 | | |  |  | 2.73 |  |
| *PRKACA* | Ssc.13872 | | |  |  | 2.42 |  |
| *MAP2K1* | Ssc.8835 | | | 1.69 |  | 1.97 |  |
| *RPS6KA3* | Ssc.4704 | | |  |  | 1.70 |  |
| *PPP3CA* | Ssc.15878 | | |  |  |  | 1.92 |
| *RAP1A* | Ssc.11026 | | |  |  |  | 2.52 |
| *RPS6KA1* | Ssc.2947 | | |  |  |  | 1.84 |
| *HRAS* | Ssc.16352 | | | 1.55 |  |  |  |
| *CALM3* | Ssc.3675 | | |  | 1.71 |  |  |
| **Metabolic Disorders** | | | | |  |  |  |
| ***Type I diabetes mellitus*** | | | | |  |  |  |
| *CPE* | Ssc.5464 | | | 2.6 |  | 3.24 |  |
| *HSPD1* | Ssc.6719 | | |  |  | 2.69 |  |
| *GAD1* | Ssc.14526 | | | | 1.51 |  |  |
| **Neurodegenerative Disorders** | | | | | |  |  |
| ***Alzheimer's disease*** | | | | |  |  |  |
| *IDE* | Ssc.9109 | | |  |  | 2.40 |  |
| *GSK3B* | Ssc.14176 | | | |  | 1.96 |  |
| ***Amyotrophic lateral sclerosis (ALS)*** | | | | | |  |  |
| *SSR4* | Ssc.2762 | | |  |  |  | 2.62 |
| *PPP3CA* | Ssc.15878 | | | |  |  | 1.92 |
| *NEF3* | Ssc.3668 | | | 4.81 |  |  |  |
| *NEFL* | Ssc.20298 | | | 2.47 |  |  |  |
| *NEFH* | Ssc.13296 | | | 1.58 |  |  |  |
| ***Dentatorubropallidoluysian atrophy (DRPLA)*** | | | | | | |  |
| *WWP1* | Ssc.1680 | | |  |  | 3.30 |  |
| *CLTC* | Ssc.7584 | | | 1.99 |  | 5.77 |  |
| *CLTA* | Ssc.11113 | | | |  | 3.18 |  |
| *CLTB* | Ssc.7163 | | | 1.62 |  | 2.58 |  |
| *DCTN1* | Ssc.11092 | | | |  | 2.24 |  |
| *CALM3* | Ssc.3675 | | |  | 1.71 |  |  |
| ***Neurodegenerative Disorders*** | | | |  |  |  |  |
| *FBXW7* | Ssc.2250 | | |  |  |  | 2.18 |
| *APLP1* | Ssc.3176 | | | 1.69 |  |  |  |
| *NEFH* | Ssc.13296 | | | 1.58 |  |  |  |
| *FBXW7* | Ssc.2250 | | |  | 2.13 |  |  |
| ***Parkinson's disease*** | | | | |  |  |  |
| *GPR37* | Ssc.27084 | | | 1.62 |  |  |  |
| ***Prion disease*** | | | |  |  |  |  |
| *NFE2L2* | Ssc.719 | | |  |  | 3.81 |  |
| *HSPD1* | Ssc.6719 | | |  |  | 2.69 |  |
| *LAMB1* | Ssc.5299 | | |  |  | 1.91 |  |
| *APLP1* | Ssc.3176 | | | 1.69 |  |  |  |
| *NFE2L2* | Ssc.719 | | | -1.63 |  |  |  |
| **Sensory System** | | | |  |  |  |  |
| ***Olfactory transduction*** | | | | |  |  |  |
| *CAMK2D* | Ssc.14480 | | | |  | 2.73 |  |
| *PRKACA* | Ssc.13872 | | | |  | 2.42 |  |
| *CALM3* | Ssc.3675 | | |  | 1.71 |  |  |
| ***Taste transduction*** | | | | |  |  |  |
| *PRKACA* | Ssc.13872 | | | |  | 2.42 |  |
| *KCNB1* | Ssc.16142 | | | 1.48 |  |  |  |
| **Signal Transduction** | | | | |  |  |  |
| ***Calcium signaling pathway*** | | | | |  |  |  |
| *PPID* | Ssc.7446 | | |  |  | 3.48 |  |
| *CAMK2D* | Ssc.14480 | | | |  | 2.73 |  |
| *PRKACA* | Ssc.13872 | | | |  | 2.42 |  |
| *ATP2A2* | Ssc.10380 | | | | 1.87* |  | 2.41* |
| *PPP3CA* | Ssc.15878 | | | |  |  | 1.92 |
| *ATP2B1* | Ssc.7985 | | |  | 1.81 |  | 2.57 |
| *ATP2A2* | Ssc.10380 | | | | 1.87 |  |  |
| *CALM3* | Ssc.3675 | | |  | 1.71 |  |  |
| ***Hedgehog signaling pathway*** | | | | |  |  |  |
| *PRKACA* | Ssc.13872 | | | |  | 2.42 |  |
| *GSK3B* | Ssc.14176 | | | |  | 1.96 |  |
| *BMP4* | Ssc.16690 | | | |  | 1.77 |  |
| ***Jak-STAT signaling pathway*** | | | | |  |  |  |
| *STAT1* | Ssc.6025 | | |  |  | 2.92 |  |
| *PTPN6* | Ssc.5530 | | |  |  | 2.10* |  |
| *PIAS4* | Ssc.47641 | | | |  | 2.09 |  |
| *SPRY2* | Ssc.45186 | | | |  | 2.01 |  |
| *MYC* | Ssc.21433 | | | |  | 1.83 |  |
| *AKT1* | Ssc.29760 | | | |  | 1.68 |  |
| *IL21* | Ssc.26323 | | | |  |  | 1.71 |
| *JAK1* | Ssc.328 | | |  |  |  | 2.24 |
| ***MAPK signaling pathway*** | | | | |  |  |  |
| *MAX* | Ssc.35004 | | | |  | 3.62* |  |
| *RAC1* | Ssc.7247 | | |  |  | 3.44 |  |
| *FOS* | Ssc.1555 | | | 1.89 |  | 2.63 |  |
| *PRKACA* | Ssc.13872 | | | |  | 2.42 |  |
| *MAP3K3* | Ssc.18489 | | | |  | 2.18 |  |
| *PPM1B* | Ssc.13719 | | | |  | 2.16 |  |
| *MAP3K7IP2* | Ssc.34309 | | | |  | 2.08 |  |
| *CDC42* | Ssc.6687 | | | 1.69 |  | 2.03* | 2.01* |
| *MAP2K1* | Ssc.8835 | | | 1.69 |  | 1.97 |  |
| *MAPKAPK5* | Ssc.1994 | | |  |  | 1.93 |  |
| *DUSP14* | Ssc.4140 | | |  |  | 1.93 |  |
| *MYC* | Ssc.21433 | | | |  | 1.83 |  |
| *HSPB2* | Ssc.19588 | | | |  | 1.80 |  |
| *DUSP6* | Ssc.25164 | | | | 1.47 | 1.79 |  |
| *GNG12* | Ssc.41291 | | | |  | 1.78 |  |
| *MAP3K4* | Ssc.4096 | | |  |  | 1.77 |  |
| *RPS6KA3* | Ssc.4704 | | |  |  | 1.70 |  |
| *AKT1* | Ssc.29760 | | | |  | 1.68 |  |
| *CACNB4* | Ssc.19686 | | | |  |  | 1.72 |
| *PPP3CA* | Ssc.15878 | | | |  |  | 1.92 |
| *GADD45A* | Ssc.20913 | | | |  |  | 2.81 |
| *RAP1A* | Ssc.11026 | | | |  |  | 2.52 |
| *MAP2K1IP1* | Ssc.13418 | | | |  |  | 2.32 |
| *RPS6KA1* | Ssc.2947 | | |  |  |  | 1.84 |
| *CACNG3* | Ssc.24475 | | | 1.82 |  |  |  |
| *HRAS* | Ssc.16352 | | | 1.55 |  |  |  |
| *CACNA2D3* | Ssc.18142 | | | | 1.58 |  |  |
| ***mTOR signaling pathway*** | | | | |  |  |  |
| *EIF4B* | Ssc.18323 | | | |  | 2.91 |  |
| *PDPK1* | Ssc.47335 | | | |  | 1.99 |  |
| *RPS6KA3* | Ssc.4704 | | |  |  | 1.70 |  |
| *AKT1* | Ssc.29760 | | | |  | 1.68 |  |
| *EIF4E2* | Ssc.18203 | | | |  |  | 1.73 |
| *RPS6KA1* | Ssc.2947 | | |  |  |  | 1.84 |
| *HIF1A* | Ssc.390 | | |  |  |  | 3.06 |
| ***Notch signaling pathway*** | | | | |  |  |  |
| *HDAC2* | Ssc.3040 | | |  |  | 2.73 |  |
| ***Phosphatidylinositol signaling system*** | | | | | | |  |
| *CALM3* | Ssc.3675 | | |  | 1.71 |  |  |
| ***TGF-beta signaling pathway*** | | | | |  |  |  |
| *PPP2CA* | Ssc.13983 | | | |  | 3.44 |  |
| *PPP2CB* | Ssc.236 | | |  |  | 3.39 |  |
| *RBX1* | Ssc.20969 | | | |  | 3.30 |  |
| *ID4* | Ssc.9645 | | | 1.75 |  | 3.21 |  |
| *ID2* | Ssc.14914 | | | |  | 2.40 |  |
| *ACVR2A* | Ssc.43549 | | | |  | 2.37 |  |
| *ID3* | Ssc.1638 | | |  |  | 2.15 |  |
| *MYC* | Ssc.21433 | | | |  | 1.83 |  |
| *BMPR2* | Ssc.33016 | | | |  | 1.78 |  |
| *BMP4* | Ssc.16690 | | | |  | 1.77 |  |
| *ACVR1* | Ssc.9902 | | |  |  | 1.76 |  |
| *THBS4* | Ssc.1411 | | |  | 1.50 |  |  |
| *PITX2* | Ssc.6994 | | |  |  |  | -1.69 |
| ***VEGF signaling pathway*** | | | | |  |  |  |
| *RAC1* | Ssc.7247 | | |  |  | 3.44 |  |
| *CDC42* | Ssc.6687 | | | 1.69 |  | 2.03* | 2.01* |
| *MAP2K1* | Ssc.8835 | | | 1.69 |  | 1.97 |  |
| *HSPB2* | Ssc.19588 | | | |  | 1.80 |  |
| *AKT1* | Ssc.29760 | | | |  | 1.68 |  |
| *PPP3CA* | Ssc.15878 | | | |  |  | 1.92 |
| *HRAS* | Ssc.16352 | | | 1.55 |  |  |  |
| ***Wnt signaling pathway*** | | | | |  |  |  |
| *RAC1* | Ssc.7247 | | |  |  | 3.44 |  |
| *PPP2CA* | Ssc.13983 | | | |  | 3.44 |  |
| *PPP2CB* | Ssc.236 | | |  |  | 3.39 |  |
| *RBX1* | Ssc.20969 | | | |  | 3.30 |  |
| *APC* | Ssc.11053 | | | |  | 3.12 |  |
| *CTNNB1* | Ssc.14003 | | | |  | 2.76 |  |
| *CAMK2D* | Ssc.14480 | | | |  | 2.73 |  |
| *PRKACA* | Ssc.13872 | | | |  | 2.42 |  |
| *GSK3B* | Ssc.14176 | | | |  | 1.96 |  |
| *MYC* | Ssc.21433 | | | |  | 1.83 |  |
| *LRP6* | Ssc.4693 | | |  |  | 1.69 |  |
| *PPP2R2A* | Ssc.104 | | |  |  | 1.64 |  |
| *PPP3CA* | Ssc.15878 | | | |  |  | 1.92 |
| *CSNK2A2* | Ssc.4909 | | |  |  |  | 1.64 |
| ***Cell adhesion molecules (CAMs)*** | | | | | |  |  |
| *PVRL3* | Ssc.27518 | | | |  | 2.96 |  |
| *CLDN7* | Ssc.825 | | | -1.49 |  | 1.93 |  |
| *ITGB1* | Ssc.4621 | | |  |  | 1.82 |  |
| *CLDN10* | Ssc.44153 | | | |  |  | 2.48 |
| *CNTN1* | Ssc.23997 | | | |  |  | 1.85 |
| *CDH2* | Ssc.22271 | | | 1.60 |  |  |  |
| *NCAM1* | Ssc.6092 | | | 2.12 | 1.67 |  |  |
| ***Cytokine-cytokine receptor interaction*** | | | | | | |  |
| *ACVR2A* | Ssc.43549 | | | |  | 2.37 |  |
| *BMPR2* | Ssc.33016 | | | |  | 1.78 |  |
| *ACVR1* | Ssc.9902 | | |  |  | 1.76 |  |
| *IL21* | Ssc.26323 | | | |  |  | 1.71 |
| *IL18* | Ssc.20 | | |  |  |  | 1.99 |
| ***ECM-receptor interaction*** | | | | |  |  |  |
| *SPP1* | Ssc.101 | | | 1.75 |  | 4.85 |  |
| *LAMB1* | Ssc.5299 | | |  |  | 1.91 |  |
| *ITGB1* | Ssc.4621 | | |  |  | 1.82 |  |
| *CD36* | Ssc.37254 | | | | -1.57 |  | 1.91 |
| *LAMA2* | Ssc.11623 | | | |  |  | 1.88 |
| *COL5A2* | Ssc.17300 | | | -1.59 |  |  |  |
| *THBS4* | Ssc.1411 | | |  | 1.50 |  |  |
| ***Neuroactive ligand-receptor interaction*** | | | | | | |  |
| *PMCH* | Ssc.3287 | | |  |  | 2.73 |  |
| *SST* | Ssc.19520 | | | 1.53 |  |  |  |

* Two or more probe sets detecting differential regulations of expression (p-value<0.01).
